# Genomic survey sequencing for development and validation of single-locus SSR markers in peanut (*Arachis hypogaea* L.)

**DOI:** 10.1186/s12864-016-2743-x

**Published:** 2016-06-01

**Authors:** Xiaojing Zhou, Yang Dong, Jiaojiao Zhao, Li Huang, Xiaoping Ren, Yuning Chen, Shunmou Huang, Boshou Liao, Yong Lei, Liying Yan, Huifang Jiang

**Affiliations:** Key Laboratory of Biology and Genetic Improvement of Oil Crops, Ministry of Agriculture, Oil Crops Research Institute of the Chinese Academy of Agricultural Sciences, Wuhan, 430062 Hubei China; Databridge Technologies Corporation, Wuhan, 430062 Hubei China

**Keywords:** Combined libraries, *de novo* assembly, Single-locus SSR, Peanut (*A hypogaea* L.)

## Abstract

**Background:**

Single-locus markers have many advantages compared with multi-locus markers in genetic and breeding studies because their alleles can be assigned to particular genomic loci in diversity analyses. However, there is little research on single-locus SSR markers in peanut. Through the *de novo* assembly of DNA sequencing reads of *A. hypogaea*, we developed single-locus SSR markers in a genomic survey for better application in genetic and breeding studies of peanut.

**Results:**

In this study, DNA libraries with four different insert sizes were used for sequencing with 150 bp paired-end reads. Approximately 237 gigabases of clean data containing 1,675,631,984 reads were obtained after filtering. These reads were assembled into 2,102,446 contigs with an N50 length of 1,782 bp, and the contigs were further assembled into 1,176,527 scaffolds with an N50 of 3,920 bp. The total length of the assembled scaffold sequences was 2.0 Gbp, and 134,652 single-locus SSRs were identified from 375,180 SSRs. Among these developed single-locus SSRs, trinucleotide motifs were the most abundant, followed by tetra-, di-, mono-, penta- and hexanucleotide motifs. The most common motif repeats for the various types of single-locus SSRs have a tendency to be A/T rich. A total of 1,790 developed *in silico* single-locus SSR markers were chosen and used in PCR experiments to confirm amplification patterns. Of them, 1,637 markers that produced single amplicons in twelve inbred lines were considered putative single-locus markers, and 290 (17.7 %) showed polymorphisms. A further F_2_ population study showed that the segregation ratios of the 97 developed SSR markers, which showed polymorphisms between the parents, were consistent with the Mendelian inheritance law for single loci (1:2:1). Finally, 89 markers were assigned to an *A. hypogaea* linkage map. A subset of 100 single-locus SSR markers was shown to be highly stable and universal in a collection of 96 peanut accessions. A neighbor-joining tree of this natural population showed that genotypes have obviously correlation with botanical varieties.

**Conclusions:**

We have shown that the detection of single-locus SSR markers from a *de novo* genomic assembly of a combination of different-insert-size libraries is highly efficient. This is the first report of the development of genome-wide single-locus markers for *A. hypogaea*, and the markers developed in this study will be useful for gene tagging, sequence scaffold assignment, linkage map construction, diversity analysis, variety identification and association mapping in peanut.

**Electronic supplementary material:**

The online version of this article (doi:10.1186/s12864-016-2743-x) contains supplementary material, which is available to authorized users.

## Background

Peanut or groundnut (*Arachis hypogaea* L.), belonging to the legume genus, is an important oil, food, and feed crop and is cultivated in more than 100 countries. The annual planting area of peanuts is approximately 21.8 Mha worldwide, with an annual production of 38.6 Mt (http://faostat.fao.org/faostat/collections?subset=agriculture 2011). The peanut production and consumption in China account for approximately 40 % of the worldwide rates, and peanut export from China has accounted for more than 30 % of the global trade since 2001 (http://zzys.agri.gov.cn/nongqing.aspx). Over 50 % of Chinese peanuts produced are crushed for extraction of oil, and peanut oil accounts for 25 % of the total domestic vegetable oil, second only to rapeseed oil. The peanut holds an important status and substantial efforts have been made to develop various types of molecular markers in recent years, such as restriction fragment length polymorphisms (RFLPs) [1, 2], random amplified polymorphic DNAs (RAPDs) [3–5], amplified fragment length polymorphisms (AFLPs) [6, 7], simple sequence repeats (SSRs) [8, 9], insertions/deletions (INDELs) [10], and single nucleotide polymorphisms (SNPs) [11, 12]. These markers were developed for genetic linkage mapping [13, 14], genetic diversity studies [9, 15, 16], and for use in plant breeding programs [10, 17]. Although many efforts have been performed by several research groups around the world, genetic research and molecular breeding of this plant lag behind those of other crops, such as rice, wheat and rape. Lack of the tools for ideal molecular markers and genomic resources are important factors hampering the development of genetic research and molecular breeding of peanut.

Single-locus markers have many advantages in molecular genetics and breeding studies compared with multi-locus markers [18–20]. The alleles of single-locus markers can be assigned to particular genomic loci in diversity analyses, preventing problems of extensive genome duplication and homology within and between different genomes caused by multi–locus markers of polyploidy [21, 22]. A series of diversity parameters can be calculated more accurately for single-locus markers than multi-locus markers, such as the number of alleles, allele frequency and polymorphism information content (PIC) [22]. Molecular markers with only a single-locus can yield accurate genotyping and are more suitable for the subsequent analysis of population structure and linkage disequilibrium (LD), while the genotyping of multi-locus markers is always ambiguous, increasing errors and making these analyses difficult.

Among the various types of molecular markers, SSRs have become the most widely used in genetic maps, gene mapping and marker-assisted selection (MAS) because of their relative abundance, good reproducibility, highly polymorphic nature, codominant inheritance pattern and random distribution in the genome [23, 24]. Based on their locations in the genome, SSR markers are generally divided into genomic SSRs and genic SSRs (or expressed sequence tag (EST)-SSRs) [25]. The usual protocol for the development of genomic SSRs has been the generation of a small-insert genomic library, subsequent hybridization with probes, and the sequencing of candidate clones [26–28]. This process is costly, technically complex, time consuming, and labor-intensive. The development of next-generation sequencing (NGS) technologies capable of quickly and inexpensively producing millions of short (50–150 bp) DNA sequence reads has prompted the use of sequence information for the identification of SSR markers [29–31]. At present, using NGS technology, the SSR markers developed are often genic SSRs based on transcriptional assemblies [32–34]. However, the genic SSRs developed are derived from coding regions that are usually conserved, leading to lower polymorphism in comparison with genomic SSRs. The development of polymorphic genic SSRs requires more experimental screening work, increasing the cost of primer synthesis and wasting resources and time. For species without a reference genome sequence, the sequencing of a combination of libraries and assembly of DNA sequences may represent an effective approach to developing markers, even single-locus SSR markers, by genome survey sequencing [35]. Combining libraries with genomic DNA inserts of different sizes, thereby randomly breaking long DNA molecules, may provide not only more complete coverage of the genome but also the necessary information for genome assembly [36, 37], because with the random positioning of fragments on the source DNA, a majority of which overlap. The development of genomic markers using this method has many advantages: it is high-throughput, fast, and results in a relatively high polymorphism rates. Markers derived from *de novo* DNA assemblies can also exhibit improved accuracy and avoid some instances of amplification failure from the transcriptome assembly due to the location of primers in intron splicing sites, which would produce primer binding sites separated by genomic introns.

Peanut is an allotetraploid (2*n* = 4 × = 40, AABB) with a large genome (~2.7 Gbp). Because of the lack of genomic information, much effort in recent years has still been focused on developing markers for peanut genetics [12, 14, 26, 38–45], with very little development of single-locus markers. In the process of constructing a consensus genetic map of the markers mapped in ten RILs and one BC mapping populations, a set of 58 single-dose SSR markers, which consistently amplified only one locus in the A or B sub-genome, was used to identify the sub-genomic origin of each linkage group, and 879 markers were eventually integrated into the map [46]. Zhou et al. [11] constructed a SNP-based linkage map that developed SNP markers using read mapping uniqueness to the consensus sequences as a filtering criterion. Consequently, the SNP markers on this map are for single loci in the AABB genome. Many existing SSR markers for the allotetraploid peanut are multi-locus because of polyploidy, and the amplified multiple fragments or loci may introduce many problems in population genetic studies. Single-locus markers can effectively avoid the issues caused by multi-locus markers. Therefore, it is attractive to develop genomic single-locus SSR markers in *A. hypogaea* for better application in genetic and breeding studies.

Here, four libraries were constructed and sequenced on the HiSeq 4000 platform. A *de novo* assembly of the DNA sequences was employed to specifically develop single-locus SSR markers in a genome-wide survey. A total of 134,652 single-locus SSR markers were developed, and their characteristics were analyzed. To validate the developed single-locus markers, some of them were evaluated by PCR-based amplification of twelve cultivated accessions, one F_2_ mapping population and one natural population.

## Results

### DNA sequencing and *de novo* genome assembly

The libraries with insert sizes of 270 bp, 500 bp, 2 Kbp and 5Kbp were sequenced with an Illumina HiSeq 4000 platform (Table 1). Massively parallel Solexa sequencing of the combination of libraries generated ~308 Gbp of raw data containing 2,056,876,970 paired-end reads, with each read being ~150 bp in length. After filtering and correction of the sequence data, a total of ~237 Gbp of clean data were obtained, with 1,675,631,984 high-quality reads and approximately 87.8 × coverage of the estimated 2.7 Gbp genome (Table 1).

**Table 1:** Summary of sequencing data

Library insert-size	No. of raw reads	Total length (bp) of raw reads	No. of high-quality reads after filtering	Total length (bp) of high-quality reads
270 bp	855,464,570	128,319,685,500	714,934,076	101,712,765,895
500 bp	543,665,696	81,549,854,400	401,580,046	55,276,499,395
2 Kbp	422,214,574	63,332,186,100	363,241,962	52,076,474,416
5 Kbp	235,532,130	35,329,819,500	195,875,900	28,017,291,974
Total	2,056,876,970	308,531,545,500	1,675,631,984	237,083,031,680

The program SOAPdenovo and all of the clean reads were used to generate a *de novo* assembly. This assembly included 2,102,446 contigs with an N50 of 1782 bp (Table 2). The majority of the contigs were in the range of 201–1000 bp (57.1 % of the contigs), and the longest contig length was 310,739 bp (Table 2). For scaffold assembly, only scaffolds greater than 200 bp in length were further analyzed. A total of 1,176,527 scaffolds were generated corresponding to 2.0 Gbp with an N50 length of 3,920 bp (Table 2). The length of the scaffolds varied from 200 bp to 576,627 bp, with an average of 1,693 bp; 360,557 scaffolds were longer than 2 Kbp and 9,448 scaffolds were longer than 10 Kbp (Table 2). The assembled genome size was ~2.0 Gbp, covering 73.6 % of the estimated 2.7 Gbp genome size. The GC content of the *de novo* assembled genome was 38.1 %.

**Table 2:** Statistics of *de novo* assembly results

	Contig		Scaffold	
	Size (bp)	Number	Size (bp)	Number
N50	1,782	-	3,920	-
Longest	310,739	-	576,627	-
Total size	1,752,933,618	-	1,987,916,087	-
Average	835	-	1,693	-
Total number	-	2,102,446	-	1,176,527
Total number (≥2 kb)	-	171,497	-	360,557
Total number (≥10 kb)	-	4,709	-	9,448

### Development and characterization of genome-wide single-locus SSR markers

The development of single-locus SSRs was based on all of the sequences from the 2.0 Gbp *de novo* genome assembly. We identified motif characters using the PERL5 script MIcroSAtellite [47] and designed primer pairs from the flanking sequences of the identified motifs using Primer3 software [48, 49]. Then, we aligned the primer pairs to the assembled scaffolds and found only one copy numbers as single-locus SSRs. Ultimately, 375,180 SSRs were found and 134,652 single-locus SSRs (Additional file 1: Table S1) were identified from them. The percentage of single-locus SSRs was 35.89 %. The frequency was 67.7 single-locus SSRs per Mb or one single-locus SSR per 14.8 Kbp. The ratio of single-locus SSRs from genetic to those from intergenetic regions was 11.2 % (13511/121141), and the ratio of non-selected SSRs from genetic to those from intergenetic regions was 14.6 % (47735/327441).

**Fig. 1:**
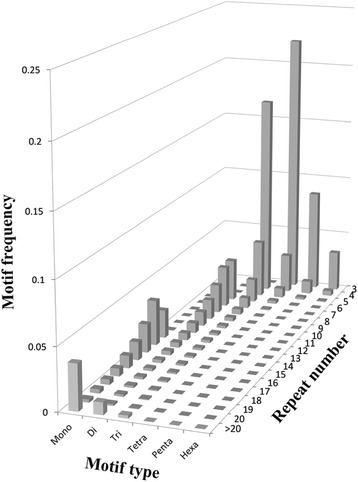
Motif frequency distributions of mono- to hexanucleotide motif types with different repeat numbers (from 3 to >20) in the *de novo* assembled genomic sequences of *A. hypogaea*

For all of the developed genomic single-locus SSR markers, a total of 155,665 motifs were found that were classified as mono- to hexanucleotide repeat types (the compound repeats were divided into corresponding nucleotide repeat types) (Table 3). The motif repeat number ranged from 3 to 146, and the repeat length was an average of 17.5 bp (Table 3). The trinucleotide repeat was the most abundant motif type, with 42,233 markers, accounting for 27.1 % of the total developed single-locus SSRs. The tetranucleotide motif also occurred at a high frequency of 26.5 %. The hexanucleotide motif had the lowest frequency of 3.9 % (Table 3). The investigation of nucleotide composition characteristics showed that A (95.1 %), AT (54.0 %), AAT (33.9 %), AAAT (37.7 %), AAAAT (29.3 %) and AAAAAT (13.7 %) were the most common motifs corresponding with the mono- to hexanucleotide repeats, respectively, suggesting that the SSRs have a tendency to be A/T rich in the peanut (Table 3). For each motif type, motif abundance decreased as the motif repeat number increased (Fig. 1). The slowest rate of decrease was for the dinucleotide motifs, and the fastest rate was for the hexanucleotide motifs (Fig. 1).

**Table 3:** The distribution of different types of single-locus SSRs identified

Motif	Number (%^1^, %^2^)	Repeat number	Total length (bp^3^, %^4^)	Average length (bp^5^)
Mono	25 224 (16.2, 100)	12–146	650 581 (23.9)	25.8
A	23 979 (15.4, 95.1)	12–146	630 491 (23.2)	26.3
C	1 245 (0.8, 4.9)	12–145	20 090 (0.7)	16.1
Di	25 460 (16.4, 100)	6–106	501 652 (18.4)	19.7
AT	13 757 (8.8, 54.0)	6–54	232 732 (8.6)	16.9
AG	8 693 (5.6, 34.1)	6–97	209 942 (7.7)	24.2
AC	2 739 (1.8, 10.8)	6–106	55 622 (2.0)	20.3
CG	271 (0.2, 1.1)	6–9	3 356 (0.1)	12.4
Tri	42 233 (27.1, 100)	4–67	672 066 (24.7)	15.9
AAT	14 329 (9.2, 33.9)	4–67	275 664 (10.1)	19.2
AAG	9 709 (6.2, 23.0)	4–49	140 004 (5.1)	14.4
AAC	3 937 (2.5, 9.3)	4–32	57 747 (2.1)	14.7
ACT	2 601 (1.7, 6.2)	4–48	38 688 (1.4)	14.9
AGT	2 586 (1.7, 6.1)	4–51	38 715 (1.4)	15.0
CCG	2 337 (1.5, 5.5)	4–9	29 151 (1.1)	12.5
AGG	2 327 (1.5, 5.5)	4–61	34 473 (1.3)	14.8
ACC	2 316 (1.5, 5.5)	4–29	31 248 (1.1)	13.5
ACG	1 069 (0.7, 2.5)	4–11	13 491 (0.5)	12.6
AGC	1 023 (0.7, 2.4)	4–16	12 885 (0.5)	12.6
Tetra	41 309 (26.5, 100)	3–43	535 760 (19.7)	13.0
AAAT	15 583 (10.0, 37.7)	3–13	205 212 (7.5)	13.2
AATT	6 035 (3.9, 14.6)	3–8	76 076 (2.8)	12.6
AAAG	5 491 (3.5, 13.3)	3–16	71 208 (2.6)	13.0
AAAC	1 949 (1.3, 4.7)	3–9	24 192 (0.9)	12.4
ACAT	1 554 (1.0, 3.8)	3–43	23 452 (0.9)	15.1
AATC	1 372 (0.9, 3.3)	3–9	17 364 (0.6)	12.7
AACT	1 319 (0.8, 3.2)	3–9	16 892 (0.6)	12.8
AATG	890 (0.6, 2.2)	3–9	11 204 (0.4)	12.6
AAGT	861 (0.6, 2.1)	3–6	10 716 (0.4)	12.4
AGAT	841 (0.5, 2.0)	3–18	11 760 (0.4)	14.0
others	5 414 (3.5, 13.1)	3–14	67 684 (2.5)	12.5
Penta	15 399 (9.9, 100)	3–10	243 955 (9.0)	15.8
AAAAT	4 505 (2.9, 29.3)	3–10	71 830 (2.6)	15.9
AAAAG	1 563 (1.0, 10.2)	3–8	24 885 (0.9)	15.9
AAATT	1 114 (0.7, 7.2)	3–6	17 355 (0.6)	15.6
AATAT	622 (0.4, 4.0)	3–8	9 825 (0.4)	15.8
AAGAT	582 (0.4, 3.8)	3–6	9 130 (0.3)	15.7
AATAG	546 (0.4, 3.5)	3–10	8 630 (0.3)	15.8
others	6 467 (4.2, 42.0)	3–8	102 300 (3.8)	15.8
Hexa	6 040 (3.9, 100)	3–20	115 266 (4.2)	19.1
AAAAAT	829 (0.5, 13.7)	3–16	15 516 (0.6)	18.7
AAAAAG	527 (0.3, 8.7)	3–7	9 906 (0.4)	18.8
AAAATT	387 (0.2, 6.4)	3–6	7 200 (0.3)	18.6
AAATAT	184 (0.1, 3.0)	3–5	3 456 (0.1)	18.8
AATCCT	182 (0.1, 3.0)	3–6	3 396 (0.1)	18.7
others	3 931 (2.5, 65.1)	3–20	75 792 (2.8)	19.3
Total	155 665 (100, −)	3–146	2 719 280 (100)	17.5

%^1^, the number of each nucleotide repeat accounted for the percentage of all motif number

%^2^, the number of each nucleotide repeat accounted for the percentage of all number of corresponding motif type

bp^3^, the total motif length of each nucleotide repeat type

%^4^, the total motif length of each nucleotide repeat type relative to the total motif length of all nucleotide repeat types

bp^5^, the total motif length of each nucleotide repeat type/the total number of nucleotide repeat types

### Validation and polymorphism detection of single-locus SSR markers in twelve inbred lines

To test whether the *in silico* developed SSR markers are single-locus, 1,790 SSR markers were selected to amplify the genomic DNA of 12 inbred lines (Additional file 2: Table S2). A total of 1,687 markers produced clear fragments, of which 1,637 (97.0 %) displayed a single amplicon, 32 (1.9 %) displayed two amplicons, and 18 (1.1 %) displayed three or more amplicons (Table 4). Of the 1,637 putative single-locus SSR markers, 290 (17.7 %) showed polymorphisms (Table 4, Additional file 2: Table S2).

**Table 4:** Amplification patterns of the 1,790 developed SSR markers in the 12 inbred lines

Primer synthesized	Amplified primers	Single amplicon	Polymorphic primers of single amplicon	Two amplicons	Three or more amplicons
1,790	1,687	1,637	290	32	18

**Fig. 2:**
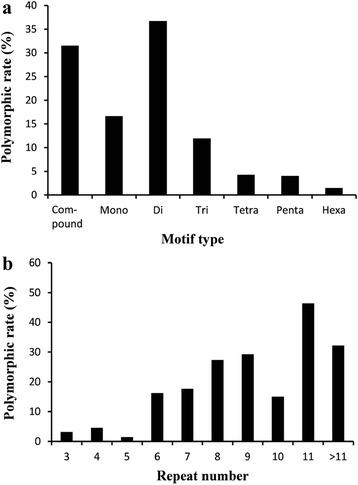
Relationship of the polymorphism rates of putative single-locus markers to the motif type (**a**) and repeat number (**b**)

We also investigated whether the motif type, repeat length and repeat number influence the polymorphism rate of single-locus SSR markers. As shown in Fig. 2a, the highest polymorphism rate was observed for the dinucleotide motifs (36.8 %), with compound motifs also showing a high rate of 31.5 %, followed by mono- (16.7 %), tri- (12.0 %), tetra- (4.3 %) and pentanucleotide motifs (4.0 %), while the lowest rate was observed for hexanucleotide motifs (1.5 %). This tendency shows that the rate of polymorphism decreases as the motif length increases, with the exception of mononucleotide motifs. No obvious relationship between the polymorphism rate and repeat length was found. Further investigations of the polymorphism rate and repeat number revealed that the maximum polymorphism rate of the developed SSR markers was 46.4 %, corresponding to a repeat number of 11. When the repeat number was less than 11, a basic trend was that the polymorphism rate tended to increase as the motif repeat number increased (Fig. 2b).

### Evaluation of inheritance and assignment of single-locus SSR markers to the linkage map

To confirm whether the developed markers amplifying a single amplicon are truly inherited in a single-locus mode, as well as to assign them to the *Arachis* linkage map, 101 high-quality markers that produced only single amplicons in the twelve inbred lines and also showed polymorphism between Zhonghua10 and ICG12625 were used for their F_2_ population survey. Of the 101 markers, 97 (96.0 %) segregated in the F_2_ population in accordance with the Mendelian inheritance law for single loci (1: 2: 1, *P* < 0.01); thus, these single-locus markers were thought to be true. Because segregation distortion is a common biological phenomenon in analyses of the genetic localizations of hybrid segregating populations [50–52], the 4 distorted markers (AHGA331177, AHGA193642, AHGA75014, AHGA84019) will be further tested for possible single-locus nature in subsequent research.

**Fig. 3:**
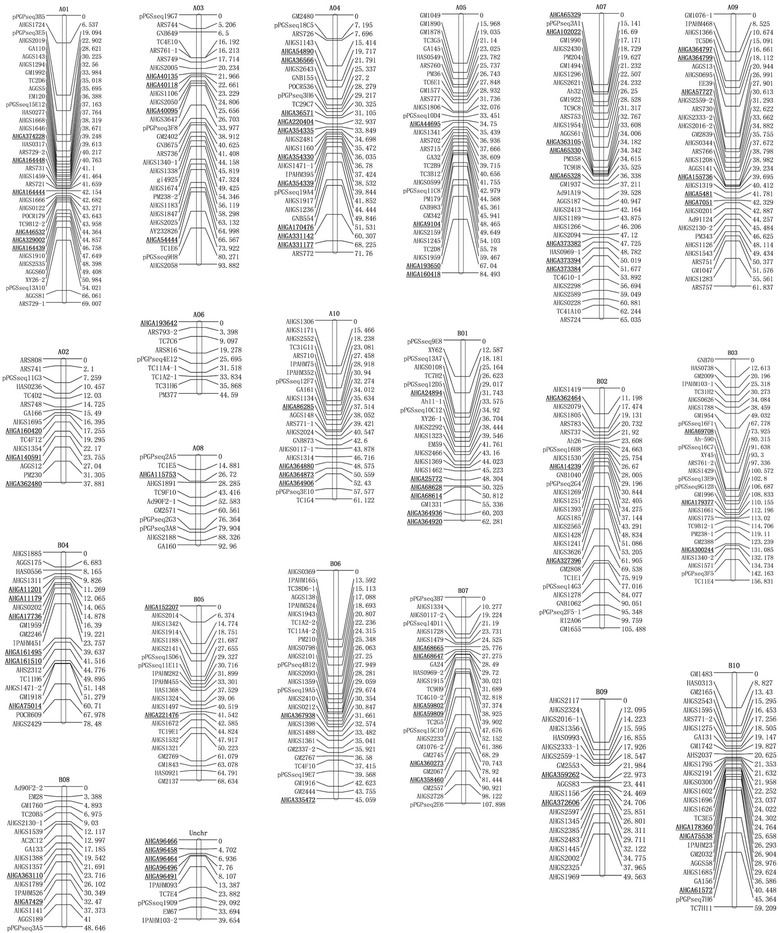
Distribution of single-locus SSR markers on the genetic linkage map. The map was constructed using 154 F_2_ plants derived from Zhonghua 10 and ICG12625. The single-locus markers developed in this study are shown in boldface and are underlined. The markers are shown on the right side of the LGs, and the map distances are shown on the left side

To assign these single-locus SSR markers to a linkage map, our previously published map for the F_2_ population derived from Zhonghua10 and ICG12625 was used as a basic frame [53]. We integrated the genotypes of these markers with previously published SSR markers. Finally, a linkage map showing the distribution of 504 SSR markers into 21 linkage groups was constructed, covering a distance of 1,504.31 cM (Fig. 3). A total of 87 (86.1 %) of the 101 single-locus SSR markers were integrated onto the map, of which 47 (54.0 %) were assigned to the A genome and 40 (46.0 %) to the B genome. The 87 single-locus markers were distributed among all of the linkage groups, with A04, at 10 single-locus markers, containing the largest number of the identified markers.

### Stability and universality of polymorphic single-locus SSR markers in *A. hypogaea*

To confirm whether the polymorphic single-locus markers tested in the 12 inbred lines are also stable and universal in more diverse lines and to test usage of the markers in DNA fingerprinting and diversity analyses, we used a population, including a set of 96 *A. hypogaea* accessions (Additional file 3: Table S3), for genotyping. A total of 100 markers were randomly selected from the polymorphic single-locus SSR markers tested in the 12 inbred lines to amplify the DNA template of this natural population, including the 4 markers with skewed segregation in the above F_2_ population. A total of 95 markers displayed single alleles in more than 95 % of the lines, 3 displayed single alleles in 90 %–95 % of the lines, and 2 displayed single alleles in 80 %–90 %. Furthermore, the observed heterozygosity (*H*_*o*_) value at each locus was calculated. The *H*_*o*_ values of the chosen SSR markers varied from 0 to 0.10 with a mean of 0.01, approaching 0 and maintaining consistency with the genomic characteristics of the inbred lines (Table 5). Among them, the *H*_*o*_ value of 74 (74 %) loci was 0, indicating that these inbred lines were homozygous at these loci. The remaining 26 markers each detected very few heterozygous lines and had a *Ho* value ranging from 0.01 to 0.10. Notably, that the 4 markers that show skewed segregation in the F_2_ mapping population all appeared as single alleles in more than 95 % of the lines, suggesting that they were also single loci. All of the selected markers appeared as single alleles in most of the *A. hypogea* accessions, except for very few multi- or null loci, suggesting that the SSR markers have a universal single-locus nature in the peanut panel.

**Table 5:** The genetic diversity of 100 SSR markers revealed by 96 *A. hypogaea* accessions

Marker	*A*	*H*_*o*_	PIC	Marker	*A*	*Ho*	PIC	Marker	*A*	*H*_*o*_	PIC
AHGA46532	8	0.00	0.70	AHGA65330	4	0.00	0.51	AHGA179378	3	0.01	0.28
AHGA164448	3	0.00	0.18	AHGA65333	6	0.01	0.65	AHGA300244	8	0.00	0.68
AHGA362464	2	0.01	0.07	AHGA65348	3	0.00	0.08	AHGA11201	5	0.01	0.51
AHGA362480	3	0.00	0.10	AHGA65349	3	0.00	0.37	AHGA11209	2	0.00	0.11
AHGA362488	2	0.00	0.02	AHGA160420	4	0.01	0.14	AHGA17736	5	0.00	0.12
AHGA362499	5	0.00	0.44	AHGA363105	7	0.01	0.60	AHGA75014	2	0.00	0.22
AHGA40095	2	0.00	0.06	AHGA373365	5	0.00	0.38	AHGA161485	2	0.00	0.06
AHGA40106	2	0.00	0.04	AHGA373382	7	0.01	0.53	AHGA161495	9	0.01	0.79
AHGA40135	8	0.04	0.54	AHGA7413	6	0.00	0.59	AHGA161510	3	0.00	0.39
AHGA54444	2	0.00	0.30	AHGA7429	8	0.01	0.68	AHGA152194	3	0.00	0.40
AHGA79898	3	0.00	0.06	AHGA164129	3	0.00	0.44	AHGA363491	3	0.00	0.54
AHGA36568	2	0.00	0.09	AHGA361225	3	0.00	0.29	AHGA363492	2	0.00	0.37
AHGA38598	4	0.00	0.20	AHGA7048	5	0.00	0.60	AHGA363495	2	0.00	0.37
AHGA38612	2	0.00	0.37	AHGA7051	4	0.00	0.35	AHGA226115	3	0.02	0.38
AHGA170476	6	0.00	0.52	AHGA22885	2	0.00	0.04	AHGA226118	2	0.00	0.09
AHGA176207	2	0.00	0.32	AHGA57727	6	0.00	0.50	AHGA59791	2	0.00	0.11
AHGA176210	2	0.08	0.21	AHGA98567	4	0.00	0.40	AHGA59797	4	0.10	0.12
AHGA220404	17	0.03	0.85	AHGA155736	3	0.00	0.12	AHGA59809	3	0.02	0.36
AHGA220933	2	0.00	0.37	AHGA75538	7	0.01	0.64	AHGA148181	2	0.00	0.11
AHGA331177	7	0.01	0.73	AHGA364906	4	0.00	0.57	AHGA244586	2	0.01	0.30
AHGA354330	12	0.01	0.77	AHGA5481	3	0.00	0.18	AHGA358460	2	0.00	0.37
AHGA354339	3	0.01	0.33	AHGA24894	10	0.00	0.75	AHGA360266	4	0.00	0.41
AHGA9097	4	0.00	0.08	AHGA25786	5	0.00	0.51	AHGA84019	11	0.00	0.86
AHGA9103	5	0.01	0.51	AHGA364915	2	0.00	0.37	AHGA352202	3	0.00	0.40
AHGA9104	5	0.00	0.57	AHGA364920	2	0.00	0.04	AHGA352262	5	0.00	0.36
AHGA44695	4	0.01	0.42	AHGA364936	6	0.00	0.52	AHGA352280	2	0.00	0.13
AHGA68628	4	0.01	0.37	AHGA14239	6	0.00	0.59	AHGA372606	18	0.00	0.81
AHGA68647	4	0.00	0.41	AHGA128473	2	0.00	0.08	AHGA61572	3	0.00	0.36
AHGA265121	4	0.00	0.48	AHGA47958	5	0.01	0.58	AHGA195525	2	0.00	0.36
AHGA362520	2	0.00	0.35	AHGA96458	4	0.00	0.54	AHGA195527	2	0.00	0.02
AHGA193642	7	0.00	0.57	AHGA96466	3	0.00	0.44	AHGA195528	2	0.00	0.04
AHGA193650	7	0.00	0.57	AHGA96491	4	0.01	0.49	AHGA214492	2	0.00	0.09
AHGA65328	2	0.00	0.35	AHGA96496	8	0.00	0.72	Mean	3.85	0.01	0.33
AHGA65329	2	0.00	0.35	AHGA159068	3	0.01	0.47	Total	428	-	-

*H*_*o*_ represents observed heterozygosity, *A* represents number of alleles, *PIC* represents polymorphism information content

To ascertain the potential value of the polymorphic single-locus markers in genetic studies, their genetic diversity in the 96 inbred lines was investigated. The 100 SSR markers generated 428 alleles (Table 5). The numbers of alleles varied from two to eighteen with a mean value of 4.28 per locus (Table 5). The PIC values of the 100 single-locus SSR markers varied from 0 to 0.86, with a mean of 0.33 (Table 5). The phylogenetic relationships of the 96 accessions were assessed using the 100 SSR markers by constructing a neighbor-joining tree (Additional file 4: Figure S1). At a similarity coefficient ≥ 0.81, the largest subgroup consisted of 39 accessions, 69.2 % of the accessions were ssp. *hypogaea* (including 23 var. *hypogaea* and 4 var. *hirsute* accessions), 15.4 % accessions were spp. *fastigiata* (including 5 var. *vulgaris* and 1 var. *fastigiata* accessions), and 15.4 % accessions were intermediate type (Additional file 4: Figure S1; Additional file 3: Table S3). The second-largest group included 31 accessions, 96.8 % of the accessions were spp. *fastigiata* (including 27 var. *vulgaris* and 3 var. *fastigiata*), and 3.2 % accessions were spp. *hypogae*a (including 1 var. *hypogaea* accession) (Additional file 4: Figure S1; Additional file 3: Table S3). At a similarity value of 0.76, a little subgroup includes 8 accessions and the number of spp. *hypogaea* and spp. *fastigiata* were each half (Additional file 4: Figure S1; Additional file 3: Table S3). In spite of a small amount of discrepancies, our results indicate that the botanical varieties of the accessions in this study obviously correspond with the genetic distances between accessions and as a result the genetic relationships among them.

## Discussion

SSRs are tandem repeats of short nucleotide motifs with a polymorphism of a certain length that are spread throughout the genome. SSRs are highly versatile, PCR-based markers that are usually associated with a high frequency of length polymorphism; thus they have a wide range of applications in genetic research and molecular breeding. However, many studies have revealed that the developed SSR markers usually amplify multiple fragments from homologous DNA sequences, because of the polyploid natures of many species [27, 54]. The multi-locus nature of SSR markers can complicate or cause errors in genotype scoring due to the reciprocal overlapping and uncertain allelism of these fragments [22]. Single-locus SSR markers can avoid this type of problem and are considered ideal markers for topics such as diversity analysis, variety identification and association analysis. Sets of high-quality single-locus SSR markers have previously been developed in plants such as potato, barley, rape, maize and grape [22, 55–58]. In our study, we developed 134,652 single-locus SSR markers for peanut. To our knowledge, this is the first report of the specific development of single-locus SSR markers in a genome-wide survey of *A. hypogaea*.

The combination of library sequencing and *de novo* assembly represents a fast and reliable approach for the generation of large datasets for peanut and also allows for the identification and development of single-locus SSRs through data mining. For assembly, the combination of libraries with different insert sizes could improve contig scaffolding much more effectively than the increasing of the physical coverage for a single insert library [59]. We generated four libraries with different insert sizes, including two libraries produced with mate pair sequencing and two short fragment insert libraries that were prepared in a separate experiment. Both ends of 150 bp reads from the four libraries could produce overlapping of the sequenced fragments and generate elongated reads. Insert sizes of 2 Kbp and 5 Kbp were more efficient than short-insert libraries (270 bp and 500 bp) because of their abilities to bridge the longer and more abundant long interspersed nuclear elements (LINE) and long terminal repeat (LTR) elements [37, 59]. The final assembly had a contig N50 value of 1,782 bp and a scaffold N50 value of 3,920 bp. The longest scaffold in the assembly was ~576.6 Kbp, and 360,557 scaffolds were longer than 2 Kbp (Table 2). The current assembly of the draft genome is 2.0 Gbp, covering 73.6 % of the estimated 2.7 Gbp total genome size. This is the first report of de novo genomic assembly of *A. hypogaea* and it can be improved by the additional sequencing of larger insert libraries to increase the contig and scaffold sizes. In addition, the data source here will contribute to genomic research of peanut.

In our study, 134,652 single-locus SSR markers were identified from 375,180 SSRs. The ratio of single-locus SSRs from genic to those from intergenic regions (11.2 %) was lower than the ratio of non-selected SSRs from genic to those from intergenic regions (14.6 %). This is probably because peanut is an allotetraploid and the genic regions are usually conserved, leading to high similarity of homoeologous genes or SSR flanking sequences in genic regions between A and B subgenomes. We developed single-locus SSR markers using only one copy numbers of primer pairs to the assembly genome scaffolds as an identification criterion. The same primer pairs in genic regions causing by homoeologous between A and B subgenomes were filtered out in our analysis.

For the developed 134,652 single-locus SSR markers, we analyzed many important characteristics. Among all of the motif types, trinucleotide repeats were the most abundant, accounting for 27.1 % of the total markers. This result may have occurred because trinucleotide repeats are just an integration of multiple codons, which do not cause frameshift mutations [60], and the prevalence of trinucleotide motifs [61] may suppress the other motif types, thus reducing the incidence of frameshift mutations caused by nontriplet repeats [62]. Interestingly, the dominant/major motifs (A, AT, AAT, AAAT, AAAAT and AAAAAT) were all A/T rich mono- to hexanucleotide motifs in peanut, which is similar to previous reports on species such as *Brassica napus*, rice, and *Arabidopsis* [54, 63, 64]. From Fig. 1, we observed that the motifs which have 3 and 4 repeats number displayed higher frequencies, 39.3 and 24.98 %, respectively. The frequency of the motifs which have 5–10 repeats number was 25.6 and > 20 repeats number had the frequency of 5.69 %. Moretzsohn et al. [14] mined 271 SSR markers in the AA genome of *Arachis* and performed a similar analysis using a two-dimensional diagram. In that study, the criteria for SSRs were different; mono- and hexanucleotide SSRs were not included; 3- and 4-repeat motifs of di- to pentanucleotide SSRs were also not included; and the product size extended to 400 bp. Therefore, markers which have 5–10-repeat motifs were most frequent, followed by > 20-repeat motifs, in contrast with the results of our survey.

Among the 1,637 selected markers that displayed a single amplicon in the twelve inbred lines, 290 (17.7 %) exhibited polymorphisms. In this study, dinucleotides motifs had higher rates of polymorphism than those with other repeat motifs, and the polymorphism rate for the single-locus SSR markers decreased as the motif length increased. In an investigation performing genome-wide SSR characterization of cucumber (*Cucumis sativus* L.), similar results were observed: dinucleotides (47 %) were the most common polymorphic motif, followed by tri- (29.3 %), tetra- (12.4 %), penta- (4.5 %), hexa- (6.9 %) [65]. This result also corresponded to the SSR mutation rates of di-, tri-, and tetranucleotide repeats in the genome of *D. melanogaster*, which found that tri- and tetranucleotide repeats mutate at rates 6.4 and 8.4 times slower than that of dinucleotide repeats, respectively [66]. In addition, we found that the polymorphism rate of the single-locus SSRs increases with increasing repeat number. Similar results have been described for several plant species [54, 67–69]. In *Brassica*, genome-wide SSR characterization showed that the polymorphism rate of the tested SSR markers was highly positively correlated with the motif repeat number (*r* = 0.74) [54]. In carrot, SSR analysis revealed a similar trend between the polymorphism rate and the repeat number; and markers containing 11–15 repeat units displayed the highest polymorphism rates [67]. This relationship is also understandable because larger motif repeat number give more opportunity for replication slippage events.

A single-locus SSR marker is revealed by a pair of oligonucleotide primers with tandem repeats of short nucleotide motifs between them and can be used in a PCR assay to detect unique site in the genome [22]. It is possible to identify these single-locus markers in DNA sequences using electronic PCR (e-PCR) by searching for subsequences of a query sequence that match the PCR primers and are in the correct order, orientation, and spacing to be consistent with the PCR product size [70, 71]. Here, using e-PCR, we identified a large number of single-locus SSRs based on the *de novo* assembled genomic sequences. Among 1,790 randomly selected *in silico* single-locus SSRs, 1,637 were able to be successfully amplified with only one band. The results demonstrate the high efficacy of e-PCR for identifying unique SSR loci in peanut.

Single-locus markers are considered to have wide utility in linkage map construction and genetic analysis of crop species due to their uniqueness. In our study, 101 high-quality SSR markers showing polymorphisms between the parental lines of Zhonghua10 and ICG12625 were experimentally confirmed as single-locus SSRs, and 89 were finally anchored in a peanut genetic map. Because these markers were located on specific chromosomes, and exhibited the characteristics of co-dominance, polymorphism and stable amplification, they can serve as anchor markers in the construction of genetic maps, thereby helping with the integration of different linkage groups. Also, polymorphism screening performed using these newly developed SSRs will greatly increase the density of SSR markers in the peanut genetic map in the future. In addition, a panel of 96 accessions was used to verify that a subset of 100 SSRs showing polymorphism and one amplicon in each of the twelve lines were genuinely single locus. These markers were further investigated for their potential use in genetic studies by ascertaining their genetic diversity in the natural population. The 100 single-locus markers generated 428 alleles with PIC values ranging from 0 to 0.86, with an average of 0.33. A set of 30 0f the 100 single-locus SSRs markers were highly informative with PIC > 0.50 (Table 5). The informative markers will be very useful to accelerate molecular genetics and breeding studies in cultivated peanut. Peanut consists of two subspecies (ssp. *hypogaea* and spp. *fastigiata*) and six botanical varieties (var. *hypogaea*, var. *hirsuta*, var. *aequatoriana*, var. *peruviana*, var. *vulgaris*, and var. *fastigiata*) that are classified based on the morphological traits of plants collected from the field [72]. Some accessions that did not belong to any of these six varieties according morphological assessment were called as intermediate varieties, because these accessions were probably generated from hybridization between different varieties. In the phylogenetic analysis of the 96 peanut accessions, the vast majority of accessions (89 %) in the two largest groups were from China, and most of exotic accessions (56.7 %) were not clustered in the two groups, suggesting the genetic basis of Chinese and exotic accessions were different. There were only one accession of var. *aequatoriana* and no accession of var. *peruviana* among the material collected. To enlarge the genetic basis, more exotic accessions should be used in future peanut breeding programs.

In many crops, genome-wide patterns of genetic variation consistently exist among different accessions [73, 74]. Studies of the seven wild relatives of soybean have revealed that approximately 80 % of the pan-genome is present in all accessions (core), whereas the rest show greater variation than the core genome, perhaps reflecting a role in adaptation to diverse environments [37]. Analysis of resequencing data of six elite maize inbred lines has revealed more than 1,000,000 SNPs, 30,000 indel polymorphisms and 101 low-sequence-diversity chromosomal intervals in the maize genome [75]. In our study, we used *de novo* assembled genomic sequences of Zhonghua 16 to design single-locus SSR markers, but a single genome does not adequately represent the diversity contained within a species. Although we used unique matching as the criterion for developing SSR markers, some markers were amplified at more than one locus in some accessions in the PCR-based experiment. Among our 1,790 validated markers, 1637 were amplified at one locus in each of the 12 lines, and 50 were amplified at more than one locus in at least one line (Table 4). In the natural population, many SSR markers displayed more than a single allele in a small number of accessions. The cause of this phenomenon may be that these loci show homeologous or heterozygous characteristics in the genomes of these accessions.

## Conclusions

In this study, we developed single-locus SSR markers by sequencing a combination of libraries and generated a *de novo* assembly of the genomic sequences of *A. hypogaea* accession Zhonghua 16. Using an e-PCR approach, 134,652 single-locus SSRs were identified by aligning primer pairs against the assembled 2.0 Gbp sequences. The validation of a set of developed markers in the twelve inbred lines, in a more diverse set of 96 accessions and in an F_2_ mapping population of 154 individuals shows the high accuracy of the developed single-locus markers. The genome wide single-locus SSR markers developed in this study will provide a useful resource for molecular markers analyses, linkage map construction, QTL mapping, and molecular breeding.

## Methods

### Library preparation and Illumina sequencing

The inbred line Zhonghua 16 was selected on the basis of its agronomic importance and the self-owned brand. The cultivar is widely grown in China and is early maturing, produces a high-yield and is resistant to drought, lodging, late leaf spot disease and rust. Short-insert (270 bp and 500 bp) and mate-pair (2 Kbp and 5Kbp) genomic DNA libraries of Zhonghua 16 were constructed. The libraries were sequenced on a llumina HiSeq 4000 platform. Using Trimmomatic 0.3 [76], low-quality, contaminant sequences were trimmed. The following types of reads were filtered: those 1) with ≥10 % unidentified nucleotides (N); 2) with >10 nt aligned to the adaptor, allowing for ≤10 % mismatches; 3) with >50 % bases having a phred quality of <5; 4) putative PCR duplicates generated by PCR amplification in the library construction process.

### *De novo* assembly

ErrorCorrection from SOAPdenovo [77] was used to connect 270-bp library paired-end reads and to generate longer sequences for assembly. Reads from all libraries were used for contig building, and 2 Kbp and 5 Kbp libraries were used to provide links for scaffold construction. GapCloser from SOAPdenovo [77] was used for gap filling within assembled scaffolds using all paired-end reads. Finally, scaffold sequences, which can be aligned to bacterial genomes with identity ≥95 % and e-value ≤1e-5, were filtered out. For identification of potential protein-coding regions in the assembly sequence we have used the gene prediction programs Fgensh [78].

### *In silico* single-locus SSR development

*In silico* single-locus SSRs that are developed should not only accord with the characteristics of SSR markers but also meet the unique characteristics of the reference genome. For the identification of SSRs, the PERL5 script MIcroSAtellite (http://pgrc.ipk-gatersleben.de/misa/) [47] was used. The motif length was defined as the default mono- to hexanucleotide, and the minimum repeat numbers of the motifs were defined as 12, 6, 4, 3, 3 and 3, respectively. For designing the primer pairs from the flanking sequences of identified SSRs, the primer3_core program (http://bioinfo.ut.ee/primer3/) was used [48, 49]. The primer design parameters were set as follows: primer length of 18–27 nucleotides, melting temperatures of 55–65 °C, GC content of 30–70 %, and predicted PCR products of 100–300 bp in length. For identification of the copy numbers, the primer pairs were aligned to the *de novo* assembly genome scaffolds of Zhonghua 16. This alignment was conducted using e-PCR [70] with the following default parameters: 2 bp mismatch, 1 bp gap, 50 bp margin and 50–1000 bp product size. The SSR markers that hit only one locus in the *de novo* assembled genome were considered single-locus SSR markers. The developed SSR markers were designated as AHGA (*Arachis hypogaea* de novo genome assembly) markers.

### DNA isolation, PCR amplification and electrophoresis

Genomic DNA was extracted from tender leaves using the modified cetyltrimethylammonium bromide (CTAB) method, essentially as described by Grattapaglia and Sederoff (1994) [79]. PCR amplification was performed in a 10 μl PCR reaction volume, containing 15 ng DNA template, 2.5 μl 2× EcoTaq PCR SuperMix and 4 pM each of the primers. PCR amplification was performed with a T100 Thermo Cycler (BIO-RAD) using the following touchdown program profile: 95 °C for 5 min; 95 °C for 30 s, 65 °C for 30 s, and 72 °C for 45 s for 9 cycles, with a reduction in the annealing temperature 1 °C per cycle; 95 °C for 30 s, 55 °C for 30 s, 72 °C for 45 s, 30 cycles; 72 °C for 5 min. The amplification products were separated by electrophoresis on 6 % denaturing polyacrylamide gels and visualized using silver-staining according to Bassam [80].

### Amplification pattern testing in 12 inbred lines, genetic localization and map construction of an F_2_ population

The randomly selected 1,790 SSR primers developed in this study were used to amplify the genomic DNA of the twelve peanut inbred lines. These lines were used as the parents of six different mapping populations (Fuchuan, ICG6375, Zhonghua10, ICG12625, Yuanza9102, Xuzhou68–4, Zhonghua6, Xuhua13, Zhonghua5, ICGV86699, Chico, Jihua9331).

The parents ‘Zhonghua10’ and ‘ICG12625’ and 154 of their F_2_ progenies were used for genetic localization. The putative single-locus SSR markers showing high quality and polymorphism between Zhonghua10 and ICG12625 were selected. Genotyping of the chosen polymorphic markers was performed on F_2_ individuals, and the allele patterns were investigated. Marker segregation was assessed with the *χ*^2^ test to examine whether they segregated as expected (1:2:1).

For the linkage map construction, input datasets were constructed from the genotypes of 101 AHGA markers in 154 F_2_ lines and integrated with the genotypes of 497 SSR markers from our previous studies [53]. The program JoinMap 4.0 [81] was used to calculate the marker order and genetic distance and the Kosambi mapping function was employed for map length estimations. The recombination frequency was set at ≤ 0.45 and LOD scores at ≥ 2.0.

### Validation of single-locus markers in a natural population

A subset of 100 developed polymorphic SSRs with one amplicon in each of the 12 inbred lines was randomly selected and a panel of 96 accessions (provided by theNational Medium-term Peanut Genebank of China) from China (66), India (24), America (5) and Zambia (1) was used for stability and diversity analyses. The genetic statistics based on the population, including the number of alleles, *H*_*O*_ and PIC, were calculated using the PowerMarker version 3.51 [82]. At a single-locus, *Ho* was determined using the following equation:

$$ Ho=1-{\displaystyle \sum_{u=1}^n}{p}_{uu} $$

in which *p*_*uu*_ is the individual frequency with homozygous allele *u*, and *n* is the number of alleles. The PIC value of individual SSR markers was calculated based on the following formula:

$$ \begin{array}{l}\mathrm{PIC}=1-\underset{i=1}{\overset{n}{\varSigma }}{p_i}^2-2\left[{\displaystyle \sum_{i=1}^{n-1}\underset{j=i=1}{\overset{n}{\varSigma }}}{p_i}^2{p_j}^2\right]\\ {}\end{array} $$

in which *pi* is the *i*th allele frequency and *n* is the number of alleles.

Coefficients of genetic similarity for the 96 cultivated accessions used in this study were calculated using the SIMQUAL program of NTSYS-pc Version 2.10 [83]. A neighbor-joining tree was constructed based on the genetic similarity matrix with the SHAN clustering program [84, 85] of NTSYS-pc using the UPGMA algorithm.

## Abbreviations

AFLP, amplified fragment length polymorphism: CTAB, cetyltrimethylammonium bromide: e-PCR, electronic PCR: *H*_*O*_, observed heterozygosities; INDEL, insertions/deletion; LD, linkage disequilibrium; LINE, long interspersed nuclear element; LTR, long terminal repeat; MAS, marker-assisted selection; NGS, next-generation sequencing; PIC, polymorphism information content; RAPD, random amplified polymorphic DNA; RFLP, restriction fragment length polymorphism; SNP, single nucleotide polymorphism; SSR, simple sequence repeat

## Additional files

Additional file 1: Table S1. Details of the identified *in silico* 134,652 single-locus SSR markers, which were determined based on the *de novo* genome assembly sequences of *A. hypogaea*. The table includes the SSR loci in the assembly sequences, the expected SSR size, the SSR type, primer and motif information. (XLSX 17082 kb) Additional file 2: Table S2. The synthesized 1,790 developed single-locus SSR markers and their amplified situation. (XLSX 121 kb) Additional file 3: Table S3. List of 96 *A. hypogaea* accessions used in this study. The table includes information about locality, accession code, subspecies and varieties. The ICRISAT codes, which can correspond to the accession code in the National Medium-term Peanut Genebank of China, are presented in parentheses. (XLSX 12 kb) Additional file 4: Figure S1. Neighbor-joining tree of the genetic relationships among 96 accessions of *A hypogaea*. The dendrogram was generated using the Jaccard similarity coefficient based on 100 polymorphic primer pairs. (JPG 539 kb)

## References

[1] Kochert G, Halward T, Branch WD, Simpson CE (1991). RFLP variability in peanut (*Arachis hypogaea* L.) cultivars and wild species. Theor Appl Genet.

[2] Kochert G, Stalker HT, Gimenes M, Galgaro L, Lopes CR, Moore K (1996). RFLP and cytogenetic evidence on the origin and evolution of allotetraploid domesticated peanut, *Arachis hypogaea* (Leguminosae). Am J Bot.

[3] Halward TM, Stalker HT, Larue EA, Kochert G (1991). Genetic variation detectable with molecular markers among unadapted germplasm resources of cultivated peanut and related wild species. Genome.

[4] Burow MD, Simpson CE, Paterson AH, Starr JL (1996). Identification of peanut (*Arachishypogaea* L.) RAPD markers diagnostic of root-knot nematode (*Meloidogynearenaria* (Neal) Chitwood) resistance. Mol Breeding.

[5] Subramanian V, Gurtu S, NageswaraRao RC, Nigam SN (2000). Identification of DNA polymorphism in cultivated groundnut using random amplified polymorphic DNA (RAPD) assay. Genome.

[6] He G, Prakash CS (1997). Identification of polymorphic DNA markers in cultivated peanut (*Arachis hypogaea* L.). Euphytica.

[7] Tallury SP, Hilu KW, Milla SR, Friend SA, Alsaghir M, Stalker HT, Quandt D (2005). Genomic affinities in Arachis section Arachis (Fabaceae): molecular and cytogenetic evidence. Theor Appl Genet.

[8] Hopkins MS, Casa AM, Wang T (1999). Discovery and characterization of polymorphic simple sequence repeats (SSRs) in peanut. Crop Sci.

[9] Macedo SE, Moretzsohn MC, Leal-Bertioli SCM, Alves DMV, Gouvea EG, Azevedo VCR, Bertiolo DJ (2012). Development and characterization of highly polymorphic long TC repeat microsatellite markers for genetic analysis of peanut. BMC Res Notes.

[10] Liu L, Dang PM, Chen CY (2015). Development and utilization of InDel markers to identify peanut (*Arachis hypogae*a) disease resistance. Frontiers Plant Sci.

[11] Zhou X, Xia Y, Ren X, Chen Y, Huang L, Huang S, Liao B, Lei Y, Yan L, Jiang H (2014). Construction of a SNP-based genetic linkage map in cultivated peanut based on large scale marker development using next-generation double-digest restriction-site-associated DNA sequencing (ddRADseq). BMC Genomics.

[12] Nagy ED, Guo Y, Tang S, Bowers JE, Okashah RA, Taylor CA, Zhang D, Khanal S, Heesacker AF, Khalilian N, Farmer AD, Carrasquilla-Garcia N, Penmetsa RV, Cook D, Stalker HT, Nielsen N, Ozias-Akins P, Knapp SJ (2012). A high-density genetic map of *Arachis duranensis*, a diploid ancestor of cultivated peanut. BMC Genomics.

[13] Bertioli DJ, Ozias Akins P, Chu Y, Dantas KM, Santos SP, Gouvea E, Guimarães PM, Leal-Bertioli SCM, Knapp SJ, Moretzsohn MC (2014). The Use of SNP Markers for Linkage Mapping in Diploid and Tetraploid Peanuts. G3(Bethesda).

[14] Moretzsohn MC, Leoi L, Proite K, Guimarães PM, Leal-Bertioli SCM, Gimenes MA, Martins WS, Valls JFM, Grattapaglia D, Bertioli DJ (2005). A microsatellite-based, gene-rich linkage map for the AA genome of *Arachis* (Fabaceae). Theor Appl Genet.

[15] Cuc LM, Mace ES, Crouch JH, Quang VD, Long TD, Varshney RK (2008). Isolation and characterization of novel microsatellite markers and their application for diversity assessment in cultivated groundnut (*Arachis hypogaea L.*). BMC Plant Biol.

[16] Moretzsohn MC, Gouvea EG, Inglis PW, Leal-Bertioli SCM, Valls JFM, Bertioli DJ (2013). A study of the relationships of cultivated peanut (*Arachis hypogaea*) and its most closely related wild species using intron sequences and microsatellite markers. Ann Bot.

[17] Pandey MK, Gautami B, Jayakumar T, Sriswathi M, Upadhyaya HD, Gowda MVC, Radhakrishnan T, Bertioli DJ, Knapp SJ, Cook DR, Varshney RK (2012). Highly informative genic and genomic SSR markers to facilitate molecular breeding in cultivated groundnut (*Arachis hypogaea*). Plant Breed.

[18] Stich B, Melchinger AE, Frisch M, Maurer HP, Heckenberger M, Reif JC (2005). Linkage disequilibrium in European elite maize germplasm investigated with SSRs. Thero Appl Genet.

[19] Comadran J, Thomas WTB, Van Eeuwijk FÁ, Ceccarelli S, Grando S, Stanca AM, Pecchioni N, Akar T, Al-Yassin A, Benbelkacem A, Ouabbou H, Bort J, Romagosa I, Hackett CA, Russell JR (2009). Patterns of genetic diversity and linkage disequilibrium in a highly structured *Hordeum vulgare* association-mapping population for the Mediterranean basin. Theor Appl Genet.

[20] Jin L, Lu Y, Xiao P, Sun M, Corke H, Bao J (2010). Genetic diversity and population structure of a diverse set of rice germplasm for association mapping. Thero Appl Genet.

[21] Chen S, Nelson MN, Ghamkhar K, Fu T, Cowling WA (2008). Divergent patterns of allelic diversity from similar origins: the case of oilseed rape (*Brassica napus* L.) in China and Australia. Genome.

[22] Li H, Younas M, Wang X, Li X, Chen L, Zhao B, Chen X, Xu J, Hou F, Hong B, Liu G, Zhao H, Wu X, Du H, Wu J, Liu K (2013). Development of a core set of single-locus SSR markers for allotetraploid rapeseed (*Brassica napus* L.). Theor Appl Genet.

[23] Morgante M, Olivieri AM (1993). PCR-amplified microsatellites as markers in plant genetics. Plant J.

[24] Powell W, Morgante M, Andre C, Hanafey M, Vogel J, Tingey S, Rafalski A (1996). The comparison of RFLP, RAPD, AFLP and SSR (microsatellite) markers for germplasm analysis. Mol Breed.

[25] Morgante M, Hanafey M, Powell W (2002). Microsatellites are preferentially associated with nonrepetitive DNA in plant genomes. Nat Genet.

[26] Shirasawa K, Koilkonda P, Aoki K, Hirakawa H, Tabata S, Watanabe M, Hasegawa M, Kiyoshima H, Suzuki S, Kuwata C, Naito Y, Kuboyama T, Nakaya A, Sasamoto S, Watanabe A, Kato M, Kawashima K, Kishida Y, Kohara M, Kurabayashi A, Takahashi C, Tsuruoka H, Wada T, Isobe S (2012). *In silico* polymorphism analysis for the development of simple sequence repeat and transposon markers and construction of linkage map in cultivated peanut. BMC Plant Biol.

[27] Lowe AJ, Moule C, Trick M, Edwards KJ (2004). Efficient large-scale development of microsatellites for marker and mapping applications in Brassica crop Species. Theor Appl Genet.

[28] Suwabe K, Iketani H, Nunome T, Kage T, Hirai M (2002). Isolation and characterization of microsatellites in *Brassica rapa* L. Theor Appl Genet.

[29] Metzker L (2010). Sequencing technologies - the next generation. Nat Rev Genet.

[30] Mardis ER (2008). The impact of next-generation sequencing technology on genetics. Trends Genet.

[31] Park I, Kim J, Lee J, Kim S, Cho O, Yang K, Ahn J, Nahm S, Kim H (2013). Development of SSR markers by next-generation sequencing of Korean landraces of chamoe (*Cucumis melo* var. *makuwa*). Mol Biol Rep.

[32] Zhang J, Liang S, Duan J, Wang J, Chen S, Cheng Z, Zhang Q, Liang X, Li Y (2012). De novo assembly and characterisation of the transcriptome during seed development, and generation of genic-SSR markers in Peanut (*Arachis hypogaea* L.). BMC Genomics.

[33] Zhang H, Wei L, Miao H, Zhang T, Wang C (2012). Development and validation of genic-SSR markers in sesame by RNA-seq. BMC Genomics.

[34] Liu Z, Chen T, Ma L, Zhao Z, Zhao PX, Nan Z, Wang Y (2013). Global transcriptome sequencing using the Illumina platform and the development of EST-SSR markers in autotetraploid alfalfa. PLoS One.

[35] Zhou W, Hu Y, Sui Z, Fu F, Wang J, Chang L, Guo W, Li B (2013). Genome Survey Sequencing and Genetic Background Characterization of *Gracilariopsis lemaneiformis* (Rhodophyta) Based on Next-Generation Sequencing. PLoS One.

[36] Sović I, Skala K, Šikić M (2013). Approaches to DNA *de novo* assembly. MIPRO.

[37] Li YH, Zhou G, Ma J, Jiang W, Jin LG, Zhang Z, Guo Y, Zhang J, Sui Y, Zheng L, Zhang SS, Zuo Q, Shi XH, Li YF, Zhang WK, Hu Y, Kong G, Hong HL, Tan B, Song J, Liu ZX, Wang Y, Ruan H, Yeung CKL, Liu J, Wang H, Zhang LJ, Guan RX, Wang KJ, Li WB, Chen SY, Chang RZ, Jiang Z, Jackson SA, Li R, Qiu LJ (2014). De novo assembly of soybean wild relatives for pan-genome analysis of diversity and agronomic traits. Nat Biotechnol.

[38] Ferguson ME, Burow MD, Schultze SR, Bramel PJ, Paterson AH, Kresovich S, Mitchell S (2004). Microsatellite identification and characterization in peanut (*A. hypogaea* L.). Theor Appl Genet.

[39] Moretzsohn MC, Hopkins MS, Mitchell SE, Kresovich S, Valls JFM, Ferreira ME (2004). Genetic diversity of peanut (*Arachis hypogaea* L.) and its wild relatives based on the analysis of hyper variable regions of the genome. BMC Plant Biol.

[40] Proite K, Leal-Bertioli SCM, Bertioli DJ, Moretzsohn MC, da Silva FR, Martins NF, Guimarães PM (2007). ESTs from a wild *Arachis* species for gene discovery and marker development. BMC Plant Biol.

[41] Wang CT, Yang XD, Chen DX, Yu SL, Liu GZ, Tang YY, Xu JZ (2007). Isolation of simple sequence repeats from groundnut. Electron J Biotechnol.

[42] Gupta PK, Kumar J, Mir RR, Kumar A (2010). Marker-assisted selection as a component of conventional plant breeding. Plant Breed Rev.

[43] Hong YB, Chen XP, Liu HY, Zhou GY, Li SX, Wen SJ, Liang XQ (2010). Development and utilization of orthologous SSR markers in *Arachis* through soybean (*Glycine max*) EST. Acta Agron Sin.

[44] Koilkonda P, Sato S, Tabata S, Shirasawa K, Hirakawa H, Saka HI, Sasamoto S, Watanabe A, Wada T, Kishida Y, Tsuruoka H, Fujishiro T, Yamada M, Kohara M, Suzuki S, Hasegawa M, Kiyoshima H, Isobe S (2012). Large-scale development of expressed sequence tag-derived simple sequence repeat markers and diversity analysis in Arachis spp. Mol Breed.

[45] Pandey MK, Monyo E, Ozias-Akins P, Liang X, Guimarães P, Nigam SN, Upadhyaya HD, Janila P, Zhang X, Guo B, Cook DR, Bertioli DJ, Michelmore R, Varshney RK (2012). Advances in *Arachis* genomics for peanut improvement. Biotechnol Adv.

[46] Gautami B, Foncéka D, Pandey MK, Moretzsohn MC, Sujay V, Qin H, Hong Y, Faye I, Chen X, BhanuPrakash A, Shah TM, Gowda MVC, Nigam SN, Liang X, Hoisington DA, Guo B, Bertioli DJ, Rami J-F, Varshney RK (2012). An international reference consensus genetic map with 897 marker loci based on 11 mapping populations for tetraploid Groundnut (*Arachis hypogaea* L.). PLoS One.

[47] Thiel T, Michalek W, Varshney RK, Graner A (2003). Exploiting EST databases for the development and characterization of gene-derived SSR-markers in barley (*Hordeum vulgare* L.). Theor Appl Genet.

[48] Untergasser A, Cutcutache I, Koressaar T, Ye J, Faircloth BC, Remm M, Rozen SG (2012). Primer3―new capabilities and interfaces. Nucleic Acids Res.

[49] Koressaar T, Remm M (2007). Enhancements and modifications of primer design program Primer3. Bioinformatics.

[50] Mangelsdorf PC, Jones DF (1926). The expression of Mendelian factors in the gametophyte of maize. Genetics.

[51] Nakagahra M (1972). Genetic mechanism on the distorted segregation of marker genes belonging to the 11th linkage group in cultivated rice. Jpn J Breed.

[52] Paterson A, Lander E, Hewitt JD, Peterson S, Lincoln SE, Tanksley SD (1988). Resolution of quantitative traits into Mendelian factors by using a complete linkage map of restriction fragment length polymorphisms. Nature.

[53] Huang L, He H, Chen W, Ren X, Chen Y, Zhou X, Xia Y, Wang X, Jiang X, Liao B, Jiang H (2015). Quantitative trait locus analysis of agronomic and quality-related traits in cultivated peanut (*Arachis hypogaea* L.). Thero Appl Genet.

[54] Shi J, Huang S, Zhan J, Yu J, Wang X, Hua W, Liu S, Liu G, Wang H (2014). Genome-wide microsatellite characterization and marker development in the sequenced Brassica crop species. DNA Res.

[55] Cipriani G, Marrazzo M, Di Gaspero G, Pfeiffer A, Morgante M, Testolin R (2008). A set of microsatellite markers with long core repeat optimized for grape (*Vitis* spp.) genotyping. BMC Plant Biol.

[56] Ghislain M, Spooner DM, Rodríguez F, Villamón F, Núñez J, Vásquez C, Waugh R, Bonierbale M (2004). Selection of highly informative and user-friendly microsatellites (SSRs) for genotyping of cultivated potato. Theor Appl Genet.

[57] Varshney R, Thiel T, Sretenovic-Rajicic T, Baum M, Valkoun J, Guo P, Grando S, Ceccarelli S, Graner A (2008). Identification and validation of a core set of informative genic SSR and SNP markers for assaying functional diversity in barley. Mol Breed.

[58] Wang FG, Tian HL, Zhao JR, Yi HM, Wang L, Song W (2011). Development and characterization of a core set of SSR markers for fingerprinting analysis of Chinese maize varieties. Maydica.

[59] van Heesch S, Kloosterman WP, Lansu N, Ruzius F-P, Levandowsky E, Lee CC, Zhou S, Goldstein S, Schwartz DC, Harkins TT, Guryev V, Cuppen E (2013). Improving mammalian genome scaffolding using large insert mate-pair next-generation sequencing. BMC Genomics.

[60] Li W, Feng Y, Sun H, Deng Y, Yu H, Chen H (2014). Analysis of simple sequence repeats in the *Gaeumannomyces graminis* var. *tritici* genome and the development of microsatellite markers. Curr Genet.

[61] Tóth G, Gáspári Z, Jurka J (2000). Microsatellites in different eukaryotic genomes: survey and analysis. Genome Res.

[62] Metzgar D, Bytof J, Wills C (2000). Selection against frameshift mutations limits microsatellite expansion in coding DNA. Genome Res.

[63] Temnykh S, DeClerck G, Lukashova A, Lipovich L, Cartinhour S, McCouch S (2001). Computational and experimental analysis of microsatellites in rice (*Oryza sativa* L.): frequency, length variation, transposon associations, and genetic marker potential. Genome Res.

[64] Katti MV, Ranjekar PK, Gupta VS (2001). Differential distribution of simple sequence repeats in eukaryotic genome sequences. Mol Biol Evol.

[65] Cavagnaro PF, Senalik DA, Yang L, Simon PW, Harkins TT, Kodira CD, Huang S, Weng Y (2010). Genome-wide characterization of simple sequence repeats in cucumber (*Cucumis sativus* L.). BMC Genomics.

[66] Schug MD, Hutter CM, Wetterstrand KA, Gaudette MS, Mackay TFC, Aquadro CF (1998). The Mutation Rates of Di-, Tri- and Tetranucleotide Repeats in *Drosophila Melanogaster*. Mol Biol Evol.

[67] Cavagnaro PF, Chung S-M, Manin S, Yildiz M, Ali A, Alessandro MS, Iorizzo M, Senalik DA, Simon PW (2011). Microsatellite isolation and marker development in carrot - genomic distribution, linkage mapping, genetic diversity analysis and marker transferability across Apiaceae. BMC Genomics.

[68] Sharopova N, McMullen MD, Schultz L, Schroeder S, Sanchez-Villeda H, Gardiner J, Bergstrom D, Houchins K, Melia-Hancock S, Musket T, Duru N, Polacco M, Edwards K, Ruff T, Register JC, Brouwer C, Thompson R, Velasco R, Chin E, Lee M, Woodman-Clikeman W, Long MJ, Liscum E, Cone K, Davis G, Coe EH (2002). Development and mapping of SSR markers for maize. Plant Mol Biol.

[69] Yi G, Lee JM, Lee S, Choi D, Kim BD (2006). Exploitation of pepper EST-SSRs and an SSR-based linkage map. Thero Appl Genet.

[70] Schuler GD (1997). Sequence mapping by electronic PCR. Genome Res.

[71] Schuler GD (1998). Electronic PCR: bridging the gap between genome mapping and genome sequencing. Trends Biotechnol.

[72] Krapovickas A, Gregory WC (1994). Taxonomía del género Arachis (Leguminosae). Bonplandia.

[73] Li YH, Li W, Zhang C, Yang L, Chang RZ, Gaut BS, Qiu LJ (2010). Genetic diversity in domesticated soybean (*Glycine max*) and its wild progenitor (*Glycine soja*) for simple sequence repeat and single-nucleotide polymorphism loci. New Phytol.

[74] Tettelin H, Masignani V, Cieslewicz MJ, Donati C, Medini D, Ward NL, Angiuoli SV, Crabtree J, Jones AL, Durkin AS, Deboy RT, Davidsen TM, Mora M, Scarselli M, Margarit Y, Ros I, Peterson JD, Hauser CR, Sundaram JP, Nelson WC, Madupu R, Brinkac LM, Dodson RJ, Rosovitz MJ, Sullivan SA, Daugherty SC, Haft DH, Selengut J, Gwinn ML, Zhou L, Zafar N, Khouri H, Radune D, Dimitrov G, Watkins K, O’Connor KJ, Smith S, Utterback TR, White O, Rubens CE, Grandi G, Madoff LC, Kasper DL, Telford JL, Wessels MR, Rappuoli R, Fraser CM (2005). Genome analysis of multiple pathogenic isolates of Streptococcus agalactiae: implications for the microbial ‘pan-genome’. Proc Natl Acad Sci U S A.

[75] Lai J, Li R, Xu X, Jin W, Xu M, Zhao H, Xiang Z, Song W, Ying K, Zhang M, Jiao Y, Ni P, Zhang J, Li D, Guo X, Ye K, Jian M, Wang B, Zheng H, Liang H, Zhang X, Wang S, Chen S, Li J, Fu Y, Springer NM, Yang H, Wang J, Dai J, Schnable PS, Wang J (2010). Genome-wide patterns of genetic variation among elite maize inbred lines. Nat Genet.

[76] Bolger AM, Lohse M, Usadel B (2014). Trimmomatic: a flexible trimmer for Illumina sequence data. Bioinformatics.

[77] Li R, Li Y, Kristiansen K, Wang J (2008). SOAP: short oligonucleotide alignment program. Bioinformatics.

[78] Salamov AA, Solovyev VV (2000). Ab initio gene finding in Drosophila genomic DNA. Genome Res.

[79] Grattapaqlia D, Sederoff R (1994). Genetic linkage maps of *Eucalyptus grandis* and *Eucalyptus urophylla* using a pseudo-testcross: mapping strategy and RAPD markers. Genetics.

[80] Bassam BJ, Caetano-Anolles G, Gresshoff PM (1991). Fast and sensitive silver staining of DNA in polyacrylamide gels. Annu Rev Plant Physiol Plant Mol Biol.

[81] van Ooijen JW (2006). JoinMap 4, Software for the calculation of genetic linkage maps in experimental populations.

[82] Liu K, Muse SV (2005). PowerMarker: an integrated analysis environment for genetic marker analysis. Bioinformatics.

[83] Rohlf FJ (2000). NTSYS-pc: Numerical Taxonomy and Multivariate Analysis System, Version 2.1.

[84] La Rota M, Kantety RV, Yu JK, Sorrells ME (2005). Nonrandom distribution and frequencies of genomic and EST-derived microsatellite markers in rice, wheat, and barley. BMC Genomics.

[85] Sneath PH, Sokal RR (1973). Numerical Taxonomy: *The Principal and Practice of Numerical Classification*.

