# Supplementary figures and images for: Genomic survey sequencing for development and validation of single-locus SSR markers in peanut (Arachis hypogaea L.)
Source: BMC Genomics. 2016 Jun 1;17:420. doi: 10.1186/s12864-016-2743-x (PMC4888616; doi:10.1186/s12864-016-2743-x)

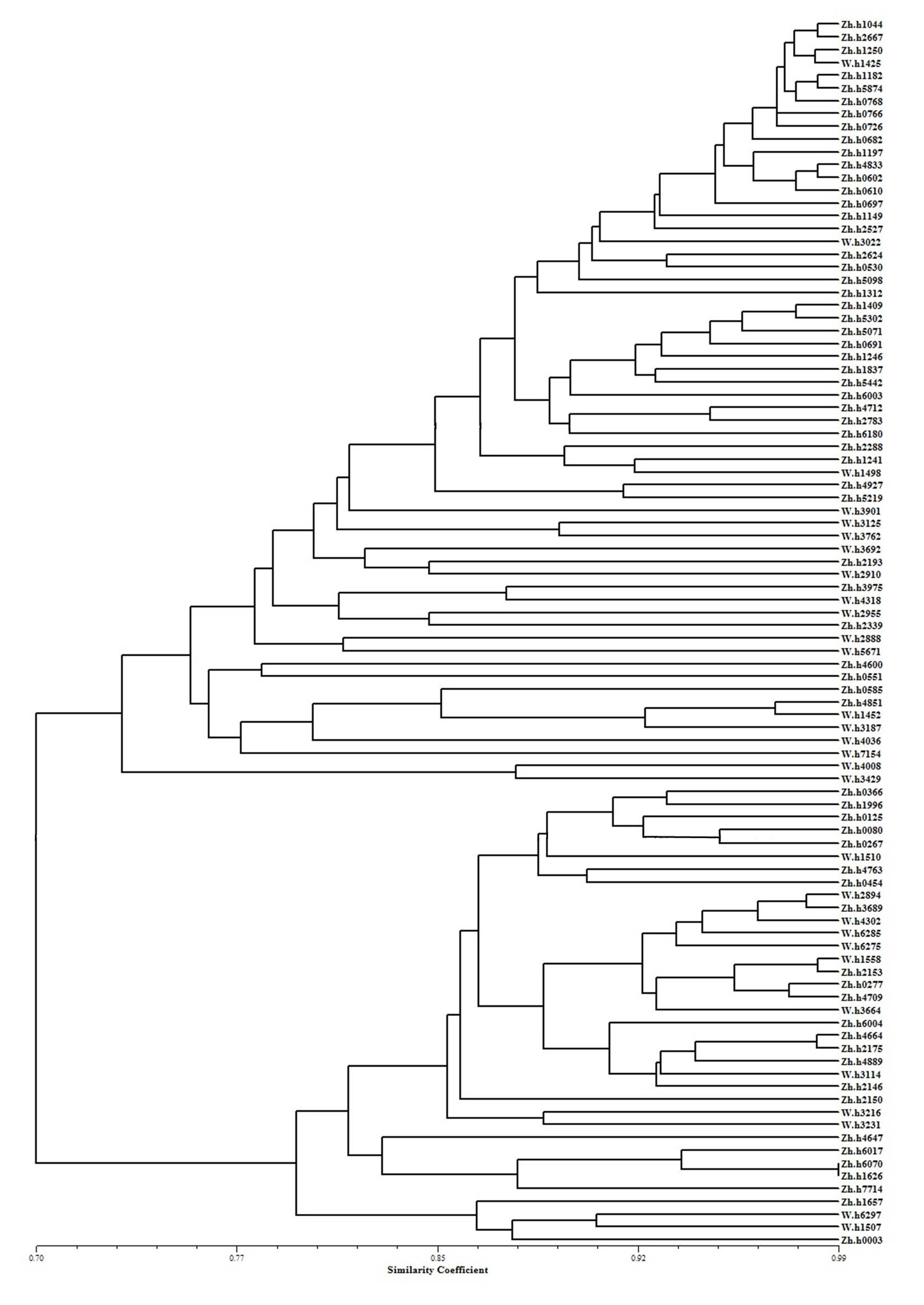

Supplement: Additional file 4: Figure S1. — Neighbor-joining tree of the genetic relationships among 96 accessions of A hypogaea. The dendrogram was generated using the Jaccard similarity coefficient based on 100 polymorphic primer pairs. (JPG 539 kb) [file 12864_2016_2743_MOESM4_ESM.jpg]
